# Case report: Leukemia cutis as the first manifestation of chronic neutrophilic leukemia in a 6-year-old girl

**DOI:** 10.3389/fped.2022.972224

**Published:** 2022-09-21

**Authors:** Ya Bin Zhou, Jia Feng Yao, Zi Gang Xu, Rui Hui Wu

**Affiliations:** ^1^Department of Dermatology, Beijing Children’s Hospital, Capital Medical University, National Center for Children’s Health, Beijing, China; ^2^Second Hematology Center, Beijing Children’s Hospital, Capital Medical University, National Center for Children’s Health, Beijing, China

**Keywords:** leukemia cutis, chronic neutrophilic leukemia, child, skin, *CSF3R*

## Abstract

Chronic neutrophilic leukemia (CNL) is a rare *BCR-ABL* negative myeloproliferative neoplasm that usually affects older adults with a poor prognosis. Leukemia cutis is an extramedullary manifestation of leukemia and may be misdiagnosed by dermatologists. Here, we describe a case of CNL in a 6-year-old Chinese girl with leukemia cutis as the first manifestation. Her skin rashes failed to attract the attention of dermatologists in early stages. The diagnosis was confirmed by peripheral smear, bone marrow studies, genomic analysis and skin biopsy.

## Introduction

Chronic neutrophilic leukemia (CNL) is a rare *BCR-ABL* negative myeloproliferative neoplasm that is characterized by neutrophilia, splenomegaly, and poor prognosis (1). CNL usually affects older adults with a median survival of approximately 2 years and only approximately 200 cases of CNL have been reported worldwide (2). Activating mutations in the colony-stimulating factor 3 receptor (*CSF3R*) gene have been identified in most cases of CNL (3). The most common mutation is T618I and has been introduced to the diagnostic criteria for CNL in the 2016 revision to the World Health Organization (WHO) classification of myeloid neoplasms and acute leukemia (4). Leukemia cutis is an extramedullary manifestation of leukemia and is associated with a worse prognosis (5). Here, we described a case of CNL in a 6-year-old Chinese girl with leukemia cutis as the first manifestation.

## Case description

**FIGURE 1:**
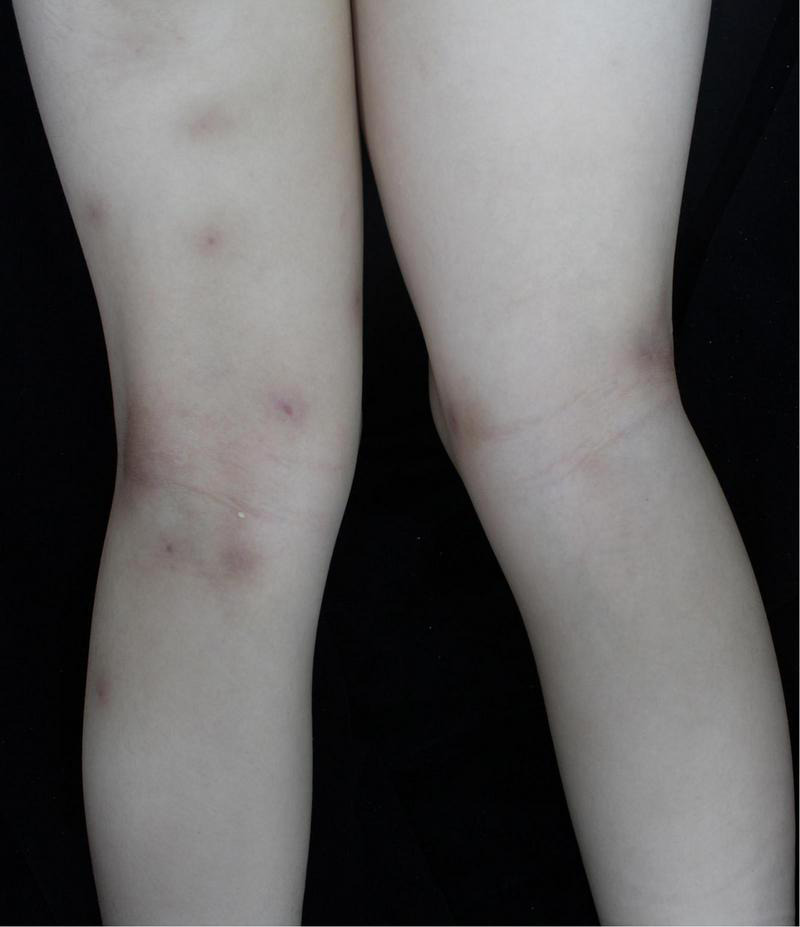
Multiple dull red plaques were observed on the lower limbs.

**FIGURE 2:**
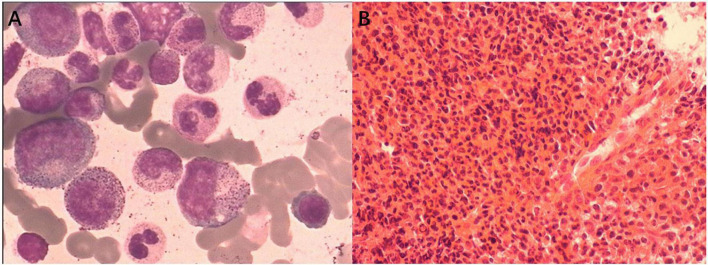
Bone marrow studies revealed hypercellular marrow with granulocytic hyperplasia. **(A)** Bone marrow smear (Wright’s stain; original magnification, ×400). **(B)** Bone marrow biospy (Hematoxylin-eosin stain; original magnification, ×100).

**FIGURE 3:**
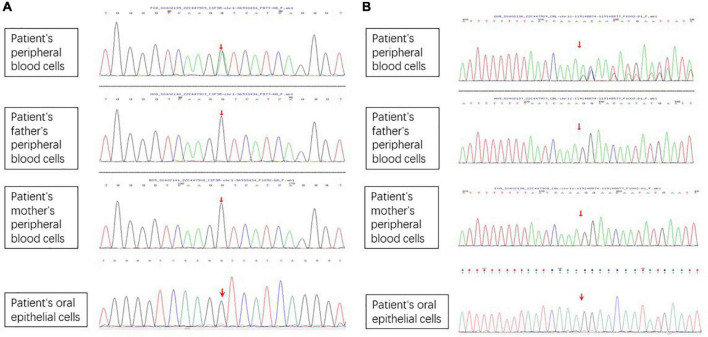
Genomic analysis revealed a CSF3R T618I somatic mutation and a CBL c.1096-1_1097delGGA frameshift somatic mutation. **(A)** CSF3R T618I somatic mutation. **(B)** CBL c.1096-1_1097delGGA frameshift somatic mutation.

**FIGURE 4:**
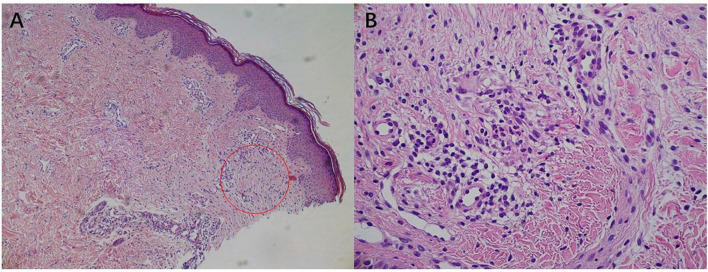
Skin biopsy showed immature blasts infiltrate. **(A)** Hematoxylin-eosin stain; original magnification, ×100. **(B)** Hematoxylin-eosin stain; original magnification, ×400.

A 6-year-old girl presented with a 10-month history of repeated cutaneous plaques and abscesses. She was initially diagnosed with furuncle, and the abscess improved after rupture. Similar rashes occurred repeatedly every 1–2 months and were resolved within 1 week. The plaques and abscesses reoccurred in the limbs 4 weeks prior. The abscesses disappeared after rupture 3 weeks prior, but arthralgia occurred in the limb joints 2 weeks prior. On physical examination, multiple dull red plaques were observed on the lower limbs (Figure 1) and splenomegaly with spleen 3 cm below the left costal margin was also noted. The complete blood count was described as follows: white blood cell count: 105.32 × 10^9^/L, neutrophils: 87.3%, lymphocytes: 9.1%, monocytes: 0.9%, eosinophils: 0.3%, basophils: 2.4%, red blood cells: 3.01 × 10^12^/L, hemoglobin: 99 g/L, and platelet count: 190 × 10^9^/L. The peripheral smear revealed a predominance of mature neutrophils with rare blasts (1%). Bone marrow studies revealed hypercellular marrow with granulocytic hyperplasia (Figure 2), negativity for *BCR/ABL* and a + 8 karyotype. Genomic analysis revealed a *CSF3R* T618I somatic mutation and a *CBL* c.1096-1_1097delGGA frameshift somatic mutation (Figure 3). Next-generation sequencing from her blood cells did not detect any other associated gene mutations. Skin biopsy revealed a few blasts infiltrated in the dermis without dermal edema (Figure 4). The blasts expressed CD33, CD68, CD117, Ki67 (5%+), and MPO but not CD2, CD3, CD30, CD56, or TdT. Then, a diagnosis of CNL with leukemia cutis was made. The patient was initially treated with hydroxyurea for 1 month. After the *CSF3R* somatic mutation was confirmed, the therapy was changed to ruxolitinib subsequently. The white blood cell count decreased to 13.16 × 10^9^/L, the skin lesions improved, and the spleen had shrunk to 1.6 cm below the left costal after 3 months treatment. She received the allogeneic hematopoietic stem cell transplantation subsequently. At present, she is in stable condition and under follow-up.

## Discussion

In CNL patients with skin lesions, sweet syndrome and leukemia cutis are difficult to differentiate because of clinical and histological similarities (6). Differentiating these conditions is important because sweet syndrome is not associated with the prognosis while leukemia cutis is a predictor of a worse prognosis (7). Sweet syndrome has an abrupt and painful onset of skin lesions which are associated with fever and improved with steroids (8). Histological examination shows a predominantly mature neutrophilic infiltrate with prominent papillary dermal edema (8). In contrast, leukemia cutis features the gradual onset of non-tender skin lesions that do not improve with steroids (8). Histological examination shows immature blasts infiltrate without significant dermal edema (8).

Chronic neutrophilic leukemia is a very rare disease and CNL with leukemia cutis is even rarer. To our knowledge, this case is the fifth case of CNL with leukemia cutis and the second case of CNL with leukemia cutis as the first manifestation. The first case of CNL with leukemia cutis as the first manifestation was presented with multiple erythematous to violaceous papules and excoriations on both lower extremities in a 70-year-old man (3). The lesions were improved with hydroxyurea and cefazolin therapy but not respond to oral steroid and empiric antibiotics therapy (3).

Chronic neutrophilic leukemia patients with leukemia cutis are considered with an aggressive course and short survival (8). In fact, the first CNL patient with leukemia cutis died 5 months after eruption (9). However, the differences between CNL patients with leukemia cutis and patients without leukemia cutis are still not well-known because of the low prevalence. Further studies should be conducted to illustrate the differences in epidemiology, treatment protocols, and survival.

The skin lesions of this patient were gradual onset plaques with abscesses and histological examination showed immature blasts infiltrate without dermal edema. These findings confirmed the diagnosis of leukemia cutis. Regretfully, skin biopsy was conducted after abscesses rupture. Therefore, only a few immature blasts were observed under histological examination. The special feature of this patient was that the leukemia cutis was the first manifestation, and it appeared far earlier than the appearance of arthralgia. She visited the dermatology department in the early stages. However, her repeated skin rashes failed to attract the attention of dermatologists. Therefore, it is necessary to remind dermatologists that chronic recurrent plaques and abscesses may be manifestations of leukemia cutis.

## Data availability statement

The original contributions presented in this study are included in the article/supplementary material, further inquiries can be directed to the corresponding author.

## Ethics statement

Written informed consent was obtained from the individual(s) and minor(s)’ legal guardian/next of kin, for the publication of any potentially identifiable images or data included in this article.

## Author contributions

RW and JY were responsible for the diagnosis and treatment of the patient. YZ and JY prepared the manuscript. RW and ZX participated in the revision of the manuscript. All authors contributed to the article and approved the submitted version.
